# Excluded futures: the continuity bias in scenario assessments

**DOI:** 10.1186/s42055-020-00030-5

**Published:** 2020-07-08

**Authors:** Paul Raskin, Rob Swart

**Affiliations:** 1 Tellus Institute, 2 Garden St, Cambridge, MA 02138 USA; 2 Wageningen Environmental Research, Droevendaalsesteeg 4, 6708 PB 9101, 6700 HB Wageningen, The Netherlands

**Keywords:** Global scenarios, Social-ecological system, Discontinuity, Vision, Socio-economic transformation, Transdisciplinary research, New paradigms

## Abstract

Global scenario assessments in support of climate, biodiversity, energy and other international policy deliberations tend to focus on a narrow bandwidth of possibilities: futures that unfold gradually from current patterns and trends. This “continuity bias” downplays the real risks (and opportunities) of structural discontinuity in the evolution of the global social-ecological system. The inclination to focus on mathematically tractable representations and conventional futures preferred by decision-makers is understandable, but constrains the scientific imagination and the scope of policy guidance. Earlier studies spotlighted discontinuous global futures, thereby revealing a broader spectrum of possibilities and repertoire of actions than found in contemporary scenario analysis. The paper revisits three types of futures introduced 25 years ago; examines three truths they convey about the contemporary moment; and points to three courses of action they suggest. Contemporary assessments centre on incrementally changing Conventional Worlds, yet varieties of global disruption (Barbarization) and progressive transformation (Great Transition) remain plausible alternatives. Corresponding to this triad, three synergistic action prongs—reform (incremental policies), remediation (emergency preparedness and prevention), and redesign (deep cultural and institutional change)—come into focus. Recovering a comprehensive perspective on the global possible would reinvigorate debate on the kind of transformation needed, broaden the action agenda, and stimulate innovative research for illuminating our indeterminate future. The COVID-19 pandemic, a concrete illustration of historical discontinuity, underscores the critical importance of emphasizing nonconventional futures in policy assessments.

## Background: spotlighting system discontinuity

The emergent interdependent global system stands as a key feature of our historical moment. Far-flung forces—environmental, economic, cultural, technological, political—are binding us together in a single community of fate. At the same time, climate change, social fissures, and other powerful stressors are eroding social-ecological resilience as we drift toward perilous thresholds of instability and discontinuity [1–3]. These unprecedented conditions demand foresight on the broad range of futures that might materialize.

Yet, international policy negotiations, and the scientific assessments that support them, have relied on a narrow bandwidth of scenarios that unfold gradually from current patterns and trends. This “continuity bias” downplays the real possibility of structural discontinuity in the evolution of the global social-ecological system. The COVID-19 pandemic is vividly illustrating one type of discontinuity. The inclination for assessments to focus on mathematically tractable representations and conventional futures preferred by decision-makers is understandable, but constrains the scientific imagination and the scope of policy guidance.

Correspondingly, policies to address urgent environmental and social problems, such as climate change, biodiversity loss, and income inequality, focus on incremental nudges to socio-economic and environmental patterns in more sustainable directions. Lessons drawn from a quarter century of visionary global scenario, paired with observations on how the world has actually unfolded, can enrich the discourse on ways to enable deep transformation and avoid collapse.

## Contrasting futures

Calls for systemic change have grown more urgent, but have a long history. The World Commission on Environment and Development [4] irrevocably etched the challenge of long-term sustainability onto the international policy agenda. In its wake, the “problem of the future” [5] drew the attention of analysts, visionaries, and activists to core existential questions: Where are we headed? Where do we want to go? How do we get there? Although the future cannot be predicted, alternative narrative and quantitative global scenarios—plausible stories about how world history might unfold in the coming decades—laid the foundation for addressing these questions.

**Fig. 1:**
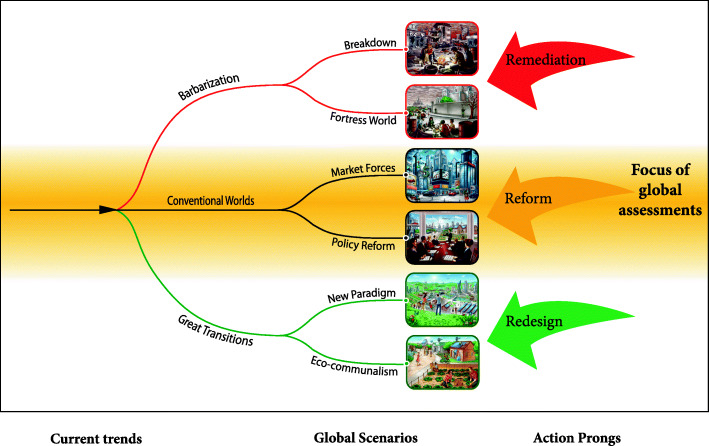
Excluded Futures and the Continuity Bias. See https://greattransition.org/explore/scenarios/excludedfutures to view an enlarged image and scenario descriptions

Responding to the challenge, a multidisciplinary, international team of natural and social scientists formed the Global Scenario Group (GSG) in 1995. The GSG organized a wide range of possible futures into three broad paths: Conventional Worlds, Barbarization, and Great Transitions [6]. These scenario categories reflect archetypal social visions—continuity, degradation, transformation—with deep roots in the history of ideas. Each scenario has two variants. Conventional Worlds assume the continuity and spread of dominant values and socio-economic patterns, driven by neoliberal policies (Market Forces variation) or, alternatively, by sustainability policies (Policy Reform). In Barbarization scenarios, Conventional Worlds crises spiral out of control, leading to an authoritarian future (Fortress World) or outright collapse (Breakdown). By contrast, Great Transitions envision responses to systemic crises of Conventional Worlds that bring forth enriched socio-economic forms, such as the autarkic Eco-Communalism variation or as a revitalized global civilization (New Sustainability Paradigm). For short descriptions and visual impressions of each scenario, please see Fig. 1.

This framework has been adopted in scores of scenario-based research studies [7, 8]. However, mainstream policy assessments, e.g., in the context of climate change, biodiversity loss and energy futures, have downplayed the possibility of collapse or structural reorganization [9, 10], thereby painting pictures of the future that generally remain within the Conventional Worlds range of possibilities,: the “continuity bias” (cf. Figure 1). Some recent analyses refer to transformational policies, but the scenarios themselves remain firmly within a Policy Reform framework [11, 12]. For climate change, high greenhouse gas emissions scenarios have been included in some analyses [13], but not the implications for the stability of global economic and natural systems. Influential policy assessments, by failing to foreground discontinuous trajectories, lack scientific rigor and imagination. The consequences are to obscure real risks, policy opportunities, and unconventional interventions.

The continuity bias may be due, at least in part, to political pressure on analysts to conform findings to the narrow outcomes acceptable to decision-makers [14]. The bias resides, as well, within the scientific discourse itself, where continuity is baked into prominent economic-environmental projection models calibrated to gradually unfolding historic trends and patterns [15]. The inadequacy of applying such mechanistic techniques to deeply uncertain global futures, akin to applying Newtonian physics to quantum phenomena, highlights the need for basic methodological innovation. For the sake of sound science, effectual policy, and better public understanding, the time is long overdue for overcoming political constraints and transcending modelling deficiencies in order to highlight the full spectrum of the global possible, from catastrophic collapse to civilizational shift.

## Which future are we living in?

Now, a quarter of a century since they were conceived, which of the GSG’s scenarios are we living in? A scan of the heterogeneous world scene reveals the answer: all of them. Global society today comprises a mosaic of Conventional Worlds, Barbarization, and Great Transition tendencies in proportions that vary across space and time [16]. Dogmatic neoliberal policy and faith in technological solutions (Market Forces) remain pervasive. At the same time, Policy Reform has emerged in widely varying degrees, e.g., in the United Nation’s Sustainable Development Goals and the Paris Agreement, and in multifarious efforts to tame the unwelcome social and environmental consequences of unbridled markets at local, national, and regional levels.

Meanwhile, multiple vectors of disruption— among them ecological disturbance, financial instability, socio-economic disparity, pandemics, and technological change—diminish social-ecological resilience, heighten migration, and unleash reactive forces. Harbingers of Barbarization lurk in rising xenophobia, chauvinism, and fundamentalism. The contemporary wave of authoritarianism could be precursor to a Fortress World, while regional chaos, conflict, state failure, and environmental calamity might presage the apocalyptic vision of Breakdown. By contrast, the same factors driving the crisis also incubate a rising cosmopolitan and ecological consciousness, antecedents of a potential Great Transition. This shift flourishes now in myriad social and environmental movements, civil society campaigns, and small-scale social experiments, all promoting cooperative economies, humane and diverse cultures, and revitalized ecosystems.

This rapidly changing melange of social forms is characteristic of a complex system approaching thresholds of systemic instability. Some observers, peering through narrow philosophical perspectives, reduce world complexity to simple truths. Celebrants of Conventional Worlds amass evidence of a world growing safer, healthier, richer [17], trusting in technological and economic responses to adequately counter the emergent crises of the growth-oriented development paradigm. Doomsayers attuned to the instabilities and inequalities that herald Barbarization warn that the end is near [18]. Paradoxically, both extremes have a case: average wealth and life expectancy have indeed risen, but so have income disparity and social fissures; local environmental remediation has transpired but macro-instabilities deepen the risk of structural rupture in biospheric processes. Transcending these polarities, novel conditions—global interdependence, shared risk, new technology—could forge another truth: the advent of a diverse transnational cultural and social movement for a Great Transition to a liveable and just future [19].

## The way ahead: three synergistic strategies

The world today evolves as a complex mixture of Conventional Worlds, Barbarization, and Great Transition tendencies. These three truths suggest three concurrent action prongs expanding on the current focus on gradual policy change: reform (incremental policy), remediation (emergency management), and redesign (system transformation). The reform prong resonates with dominant policy paradigms seeking to ease social-ecological stress, such as cautious efforts to control greenhouse gas emissions. Unfortunately, conventional institutions, notably the state-centric international order and corporate dominated political economy, appear profoundly ill-equipped to meet the challenge of deep reform. The most promising efforts, such as the Paris Agreement on climate change and the United Nations Sustainable Development Goals, are steps in the right direction, but not the leap forward now needed. Still, civil society reform efforts can help mute dangerous trends, thereby countering Barbarization while buying time for a Great Transition mobilization. However, evidence mounts that incremental action alone is insufficient, especially as key government and corporate leaders continue to deny, ignore, or respond indecisively to threats.

The second action prong—emergency management—counters head-on the real risk of system collapse (Barbarization). This strategy evokes an existential “precautionary principle” proscribing policies that allow further drift toward conditions where science cannot rule out social-ecological tipping points. It would be timely to extend the environmental precautionary principle, embodied in Principle 15 of the 1992 United Nations Conference on Environment and Development Rio Declaration [20] to the system level. Additionally, redoubled cultural and educational efforts are needed to counter the politics of hate and polarization. In parallel, international emergency preparation for humane intervention into hotspots of chaos and conflict are essential, lest military containment becomes the rule. Finally, critical consideration of selected geoengineering options compatible with the precautionary principle, such as massive biomass sequestration in soils, rather than perilous solar radiation management, would be prudent.

Since thus far the reform and emergency prongs have proved too little, too late, the third prong comes to the fore: actions to advance transformative cultural and institutional change. A robust strategy for deep change has many dimensions, including designing innovative economic and governance models attuned to contemporary challenges, debating alternative global visions, and nurturing a shift toward values of global solidarity, ecological sensibility, and lives of qualitative fulfilment over consumerism. Critical to this approach are new initiatives to foster connectivity across popular movements and civil society networks, thereby creating a path to an overarching movement of global citizens for a Great Transition.

The three action prongs—reform, remediation, and redesign—are best pursued synergistically, rather than as independent strategies. Non-government actors and networks are critical to all dimensions: prodding governmental reform, prompting calamity control, and galvanizing transformative movements. In parallel, research can better support and guide these efforts by giving priority to the exploration of nonconventional futures and their links to near term choices. For example, for climate, integrated assessment models used to quantify greenhouse gas emissions underlying climate projections do neither incorporate the potentially disruptive feedbacks of climate impacts on economic and demographic drivers of emissions, nor are they equipped to deal with deep societal or economic transformation. Most immediately, assessments such as those of the IPCC need to be enhanced to incorporate disruptive change, whether the feedbacks of severe climate change on economic and demographic assumptions or the impacts of a deep shift in human values and institutions.

Beyond the climate issue, the search for pathways to social-ecological sustainability requires integrated analysis across sectors, geographic scales, and time horizons. The research agenda now taking shape to address this challenge [21] would be well advised to highlight the exploration of system discontinuity and transformation as a critical dimension for deepening understanding, broadening policy, and engaging citizens. Facing a holistic challenge, we need a new transdisciplinary science that, in collaboration with artists, historians, innovators and social visionaries, can propel awareness and action by illuminating the landscape of the future, in all its dire peril and unique opportunity. This would better connect science, policy and society, and foster explorations of alternative paradigms for a civilization fit for the twenty-first century. The COVID-19 pandemic has painfully demonstrated the real risk of historical discontinuity. A varied array of other social-ecological discontinuities can plausibly emerge in the coming decades. Going forward, scenario assessments with claims to relevance and rigor must emphasize nonconventional global futures.

## Data Availability

Not applicable.

## References

[1] Rockström J, Steffen W, Noone K, Persson Å, Chapin FSIII, Lambin E, et al. Planetary boundaries: exploring the safe operating space for humanity. Ecol Soc. 2009;14(2):32.

[2] Carpenter SR, Folke C, Scheffer M, Westley FR. Dancing on the volcano: social exploration in times of discontent. Ecol Soc. 2019;24(1):23.

[3] WEF (World Economic Forum). The global risks report 2020. Davos: World Economic Forum; 2020.

[4] Brundtland GH. Our common future. Geneva: Report of the world commission on environment and development; 1987. UN-document A/42/427.

[5] Swart RJ, Raskin P, Robinson J. The problem of the future: sustainability science and scenario analysis. Glob Environ Chang. 2004;14:137–46.

[6] Raskin P, Banuri T, Gallopin G, Gutman P, Hammond A, Kates R, et al. Great transition: the promise and lure of the times ahead. Boston: Stockholm Environment Institute; 2002. https://www.tellus.org/tellus/publication/great-transition-the-promise-and-lure-of-the-time-ahead.

[7] Hunt DVL, Lombardi DR, Atkinson S, Barber ARG, Barnes M, Boyko CT, et al. Scenario archetypes: converging rather than diverging themes. Sustainability. 2012;4(4):740–72.

[8] UNEP (United Nations Environment Programme). Global environment outlook 3 - past, present and future perspectives. London/Sterling: Earthscan Publications Ltd; 2002.

[9] IPCC (intergovernmental panel on climate change). In: Nakicenovic N, Swart R, editors. Special report on emissions scenarios. England: Cambridge University press; 2000.

[10] IEA (International Energy Agency). World energy outlook 2018. Paris: International Energy Agency; 2018.

[11] Riahi K, van Vuuren DP, Kriegler E, Edmonds J, O’Neill BC, Fujimori S, et al. The shared socioeconomic pathways and their energy, land use, and greenhouse gas emissions implications: an overview. Glob Environ Chang. 2016;42:153–68.

[12] IPBES (intergovernmental science-policy platform on biodiversity and ecosystem services). Summary for policymakers of the global assessment report on biodiversity and ecosystem services – advance unedited version; 2019.

[13] Riahi K, Rao S, Krey V, Cho C, Chirkov V, Fischer G, et al. RCP 8.5—a scenario of comparatively high greenhouse gas emissions. Clim Change. 2011;109:33. 10.1007/s10584-011-0149-y.

[14] Hausfather Z, Peters GP. Emissions – the ‘business as usual’ story is misleading. Nature. 2020;577:618–20. 10.1038/d41586-020-00177-3. 31996825 10.1038/d41586-020-00177-3

[15] Anderson K, Jewell J. Debating the bedrock of climate-change mitigation scenarios. Nature. 2019;573:348–9. 31527780 10.1038/d41586-019-02744-9

[16] Gallopín G. Back to the future energy policy 123; 2018. p. 318–24.

[17] Rosling H, Rönnlund AR, Rosling O. Factfulness: ten reasons We're wrong about the world – and why things are better than you think. New York: Flatiron Books; 2018.

[18] Wallace-Wells D. The uninhabitable earth: life after warming. New York: Tim Duggan Books; 2019.

[19] Raskin P. Journey to Earthland: the great transition to planetary civilization. Boston: Tellus Institute; 2016. https://www.tellus.org/tellus/publication/journey-to-earthland.

[20] UNCED (United Nations Conference on Environment and Development). Rio declaration on environment and development; 1992. UN Doc. A/CONF.151/26 (vol. I); 31 ILM 874.

[21] Köhler J, Geels FW, Kern F, Markard J, Onsongo E, Wieczorek A, et al. An agenda for sustainability transitions research: state of the art and future directions. Environ Innov Societal Trans. 2019;31(2019):1–32. 10.1016/j.eist.2019.01.004.

